# How intervention studies measure the effectiveness of medication safety-related clinical decision support systems in primary and long-term care: a systematic review

**DOI:** 10.1186/s12911-024-02596-y

**Published:** 2024-07-04

**Authors:** David Lampe, John Grosser, Dennis Grothe, Birthe Aufenberg, Daniel Gensorowsky, Julian Witte, Wolfgang Greiner

**Affiliations:** 1 Department of Health Economics and Health Care Management, School of Public Health, Bielefeld University, Universitätsstraße 25, Bielefeld, 33615 Germany; 2 Vandage GmbH, Bielefeld, Germany

**Keywords:** Systematic review, Medical Order Entry systems, Decision support systems, clinical, Outcome Assessment (Health Care), Medication errors, Primary Health Care

## Abstract

**Background:**

Medication errors and associated adverse drug events (ADE) are a major cause of morbidity and mortality worldwide. In recent years, the prevention of medication errors has become a high priority in healthcare systems. In order to improve medication safety, computerized Clinical Decision Support Systems (CDSS) are increasingly being integrated into the medication process. Accordingly, a growing number of studies have investigated the medication safety-related effectiveness of CDSS. However, the outcome measures used are heterogeneous, leading to unclear evidence. The primary aim of this study is to summarize and categorize the outcomes used in interventional studies evaluating the effects of CDSS on medication safety in primary and long-term care.

**Methods:**

We systematically searched PubMed, Embase, CINAHL, and Cochrane Library for interventional studies evaluating the effects of CDSS targeting medication safety and patient-related outcomes. We extracted methodological characteristics, outcomes and empirical findings from the included studies. Outcomes were assigned to three main categories: process-related, harm-related, and cost-related. Risk of bias was assessed using the Evidence Project risk of bias tool.

**Results:**

Thirty-two studies met the inclusion criteria. Almost all studies (*n* = 31) used process-related outcomes, followed by harm-related outcomes (*n* = 11). Only three studies used cost-related outcomes. Most studies used outcomes from only one category and no study used outcomes from all three categories. The definition and operationalization of outcomes varied widely between the included studies, even within outcome categories. Overall, evidence on CDSS effectiveness was mixed. A significant intervention effect was demonstrated by nine of fifteen studies with process-related primary outcomes (60%) but only one out of five studies with harm-related primary outcomes (20%). The included studies faced a number of methodological problems that limit the comparability and generalizability of their results.

**Conclusions:**

Evidence on the effectiveness of CDSS is currently inconclusive due in part to inconsistent outcome definitions and methodological problems in the literature. Additional high-quality studies are therefore needed to provide a comprehensive account of CDSS effectiveness. These studies should follow established methodological guidelines and recommendations and use a comprehensive set of harm-, process- and cost-related outcomes with agreed-upon and consistent definitions.

**Prospero registration:**

CRD42023464746

**Supplementary Information:**

The online version contains supplementary material available at 10.1186/s12911-024-02596-y.

## Introduction

Medication errors are a common problem in health care and a frequent cause of mortality and morbidity [1–3]. Due to inconsistent definitions and classification systems, differences in populations studied and varying outcome measures, the reported prevalence of medication errors and adverse drug events (ADE) varies widely (from 2% to 94%) across different studies [1, 2, 4–6]. Given the high number of prescriptions in primary care, medication errors have the potential to cause considerable harm [7–9], contributing to substantial health and economic consequences, including an increased utilization of health care services and, in the worst case, patient death [10–12].

The use of digital health technologies can help overcome shortcomings at each stage of the medication management process [13]. Digital health technologies have the potential to reduce medication errors and adverse drug reactions (ADR), improve patient safety and thus contribute to higher quality and efficiency in health care [14, 15]. In particular, Clinical Decision Support Systems (CDSS) are used to improve medication safety by providing direct medication related advice to physicians, pharmacists or other participants involved in the medication process [16, 17]. Current research demonstrates the potential of CDSS to enhance health care processes [18–23]. In particular, CDSS that are integrated into the clinical workflow and include messages or alerts that are automatically presented during clinical decision making can have beneficial effects [24].

While a variety of studies have examined the effects of CDSS on medication safety, significant heterogeneity exists concerning the outcome measures used, leading to an ambiguous body of evidence [16, 25, 26] – particularly in primary care [27–29] and long-term care (LTC) [29–31]. According to Seidling and Bates [32], outcomes used by studies investigating the impact of digital health technologies on medication safety can be grouped into three categories: process-related, harm-related, and cost-related outcomes. These categories differ regarding their relevance for patient health [32]. In particular, harm-related outcomes are more directly relevant for patient health than process- or cost-related outcomes.

As of yet, no review has comprehensively summarized the outcome measures used in studies on medication safety-related CDSS effectiveness in primary care and LTC. Therefore, the primary objective of this systematic review is to summarize and categorize the outcome measures used in these studies. Thereby, we contribute to a more standardized approach in the evaluation of CDSS and facilitate future research in this field. A secondary aim is to compare the main empirical findings of these studies.

## Methods

Our systematic review followed the guidelines outlined in the Preferred Reporting Items for Systematic Reviews and Meta-Analyses (PRISMA 2020) Statement [33] (see Supplementary Tables S1-S2, Additional File 1). This systematic review was registered with PROSPERO (CRD42023464746) [34].

### Search strategy

We systematically searched PubMed, Embase, CINAHL, and the Cochrane Library for papers published before September 20^th^, 2023. The search strategy included terms about the character and type of intervention (digital decision support), the aim of these interventions (medication safety) and the targeted setting (outpatient/primary and LTC). Relevant MeSH-terms were considered (see Supplementary Table S1, Additional File 2). We developed the search strategy in accordance with published CDSS-related systematic reviews [25, 26, 28, 35]. Further publications were searched manually via hand search and automatically using forward and backward citation of the Spider Cite tool [36].

### Eligibility criteria

We included English and German language full-text publications that report data on interventional studies evaluating CDSS to improve the medication safety in the primary/outpatient and LTC setting. Only studies reporting medication-, patient- or cost-related outcomes were included, while studies reporting only outcomes related to healthcare providers attitude or acceptance regarding CDSS and studies focusing only on performance or quality indicators of CDSS (e.g. sensitivity, specificity) were excluded. Studies were also excluded if the intervention was conducted in inpatient care, did not automatically engage in the medication process (e.g., via automated alerts), or included only a simple reminder function. Furthermore, studies were not eligible if they focused only on a single potentially problematic drug or only on one specific indication. Finally, studies were excluded if they did not primarily aim at the improvement of medication safety. There were no restrictions regarding the comparator of the intervention (see Supplementary Table S2, Additional File 2). Two investigators (DL and DGR) independently screened search results and assessed the eligibility of potentially relevant studies according to the predefined inclusion and exclusion criteria. Discrepancies (*n* = 131) were resolved by consensus. Another investigator (BA) was consulted if consensus could not be reached.

### Data extraction, categorization and synthesis

We extracted the following data from the included studies: study design, study period, sample, and setting, type of intervention and comparator (Table 1), primary and secondary outcome measures (Table 2), outcome levels (Table 3), and main empirical findings (Table 4). Two investigators (DGR, JG) jointly performed the data extraction, which was verified by a third investigator (BA). We grouped types of interventions and comparators into the following categories:

**Table 1:** Study characteristics

Study	Country	Design	Period (months)	Sample (*n*)	Setting	Intervention	Control
Abramson et al., 2011a [37]	USA	N-RCT	12	2,866	**PCP** 11 practices, 21 providers	EHR + eRx + CDSS	pRx
Abramson et al., 2011b [38]	USA	PPS	12	2,096	**OC** 17 providers	**Pre**: EHR + CPOE + CDSS **Post**: EHR + CPOE + eRx + CDSS	n.a.
Abramson et al., 2013 [39]	USA	PPS	24	3,158	**OC** 24 providers	**Pre**: EHR + CPOE + CDSS **Post**: EHR + CPOE + eRx + CDSS	n.a.
Andersson et al., 2013 [40]	SWE	N-RCT	8^a^	50,017	**PCP** 20 health care centers	**Pre**: EHR + eRx + CDSS (old) **Post**: EHR + eRx + CDSS (with new DDI-database)	EHR + eRx + CDSS (old)
Field et al., 2009 [41]	CAN	C-RCT	12	833	**LTCF** 22 long-stay units, 10 physicians	EHR + CPOE + CDSS	EHR + CPOE
Glassman et al., 2007 [42]	USA	RCT	12^a^	913	**OC** Medical provider with several OC	EHR + CPOE + CDSS + DUR	EHR + CPOE + CDSS
Gurwitz et al., 2008 [43]	CAN USA	C-RCT	6, 12 (2 sites)	1,118	**LTCF** 2 LTCF, 29 resident care units, 37 prescribers	CPOE + CDSS	CPOE
Hou et al., 2013 [44]	TWN	PPS	6^a^	2,357	**OC** Hospital with OC	**Pre**: CPOE **Post**: CPOE + CDSS	n.a.
Humphries et al., 2007 [45]	USA	PPS	57	555	**HMO** 18 medical offices, pharmacies	**Pre**: PIMS + passive CDSS **Post**: PIMS + CDSS	n.a.
Jani et al., 2008, UK [46]	UK	PPS	13^a^	520	**OC** Hospital with pediatric nephrology OC	**Pre**: pRx **Post**: eRx + CDSS	n.a.
Judge et al., 2006 [47]	USA	C-RCT	12	445	**LTCF** 7 resident care units, 27 prescribers	CPOE + CDSS	CPOE
Jungo et al., 2023 [48]	CHE	C-RCT	12	323	**PCP** 43 GP	EHR + CDSS	EHR
Kahan et al., 2017 [49]	ISR	N-RCT	18^a^	32,943	**HMO** 715 primary care physicians	EHR + CDSS	EHR
Kaushal et al., 2011 [50]	USA	N-RCT	12	3,720	**PCP** 11 practices, 21 providers	eRx + CDSS	EHR + eRx + less robust CDSS
Kaushal et al., 2010 [51]	USA	N-RCT	12	10,711	**PCP** 12 practices, 30 providers	**Pre**: pRx **Post**: eRx + CDSS	**Pre**: pRx **Post**: pRx
Mazzaglia et al., 2016 [52]	ITA	C-RCT	24	25,491	**PCP** 197 GP	Standard software + CDSS	Standard software + paper-based information
Overhage et al., 2016 [53]	USA	PPS	12	103,009	**PCP** 2 study sites, 17 practices, 228 physicians	**Pre**: (study site 1) pRx **Pre**: (study site 2) eRx **Post**: EHR + eRx + CDSS	n.a.
Price et al., 2017 [54]	CAN	C-RCT	8^a^	9,467	**PCP** 8 practices, 28 physicians	EHR + CDSS (with STOPP rules alerts)	EHR + CDSS (without STOPP rules alerts but other alerts)
Raebel et al., 2007a [55]	USA	RCT	4	11,100	**HMO** Medical offices, pharmacies	PIMS (EHR + CPOE) + CDSS + physician consultation	PIMS (EHR + CPOE)
Raebel et al., 2007b [56]	USA	RCT	12	59,680	**HMO** 426 prescribers, 18 medical offices, 21 pharmacies	PIMS (EHR + CPOE) + CDSS + physician consultation	PIMS (EHR + CPOE)
Rieckert et al., 2020 [57]	GER	C-RCT	24	3,904	**PCP** 359 practices	EHR + CDSS	Treatment as usual
Schwarz et al., 2012 [58]	USA	C-RCT	28^a^	35,111	**PCP** 2 practices, 41 providers	EHR + CDSS	EHR + CDSS
Simon et al., 2006 [59]	USA	C-RCT	41^a^	50,924	**HMO** 15 practices, 239 clinicians	EHR + CPOE + CDSS + Education	EHR + CPOE + CDSS
Smith et al., 2006 [60]	USA	PPS	39	approx. 450,000	**HMO** 15 clinics, 209 providers	**Pre**: EHR + CPOE **Post**: EHR + CPOE + CDSS	n.a.
Steele et al., 2005 [61]	USA	PPS	9	19,076	**OC** approx. 120 providers	**Pre**: EHR + CPOE **Post**: EHR + CPOE + CDSS	n.a.
Subramanian et al., 2012 [62]	CAN	C-RCT	12	833	**LTCF** 22 long-stay units, 10 physicians	EHR + CPOE + CDSS	EHR + CPOE
Tamblyn et al., 2012 [63]	CAN	C-RCT	23^a^	5,628	**PCP** 81 physicians	EHR + CDSS (with risk of injury alert)	EHR + CDSS (without risk of injury alert)
Tamblyn et al., 2008 [64]	CAN	C-RCT	18^a^	3,449	**PCP** 28 physicians	EHR + eRx + CDSS (automatic)	EHR + eRx + CDSS (on-demand)
Tamblyn et al., 2003 [65]	CAN	C-RCT	13	12,560	**PCP** 107 physicians	EHR + CDSS + health-problem list	EHR + health-problem list
Vanderman et al., 2017 [66]	USA	PPS	24^a^	3,029	**OC** Medical center with OC	**Pre**: EHR + CPOE **Post**: EHR + CPOE + CDSS	n.a.
Witte et al., 2019 [67]	GER	PPS	18^a^	874	**PCP** 15 physicians	**Pre**: Standard software^b^ **Post**: EHR + CDSS	n.a.
Zillich et al., 2008 [68]	USA	PPS	12^a^	2,753	**OC** 8 hospitals, 28 OC	**Pre**: EHR + CDSS **Post/1st stage**: EHR + CDSS (with alerts for five selected high-risk drugs) **Post/2nd stage**: Handwritten advise and education materials	n.a.

*CAN *Canada, *CDSS *Clinical decision support system, *CHE *Switzerland, *CPOE *Computerized provider order entry, *C-RCT *Cluster-randomized controlled trial, *EHR *Electronic health record, *eRx *Electronic (e-)prescribing, *GER *Germany, *GP *General practitioner, *HMO *Health maintenance organization, *ISR *Israel, *ITA *Italy, *LTCF *Long-term care facility, *n.a.* Not applicable, *N-RCT *Non-randomized controlled trial, *OC *Outpatient clinic, *PCP *Primary care practice, *PIMS *Pharmacy information management system, *PPS *Pre-post study, *pRx *Paper-based/handwritten prescribing, *RCT *Randomized controlled trial, *SWE *Sweden, *TWN *Taiwan, *UK *United Kingdom, *USA *United States of America

^a^duration calculated/converted by the authors of this study

^b^no further information provided

**Table 2:** Overview of extracted primary and secondary outcomes including outcome (sub-)categories and levels of operationalization

Study	Outcomes	Outcome category (subcategory)	Level of operationalization
Abramson et al., 2011a [37]	Error-associated (preventable) ADE	harm-related (injuries)	prescription-level
	Prescribing errors Near misses Rule violations	process-related (error rate)	prescription-level
Abramson et al., 2011b [38]	Error-associated (preventable) ADE	harm-related (injuries)	prescription-level
	Prescribing errors Near misses Rule violations	process-related (error rate)	prescription-level
Abramson et al., 2013 [39]	Error-associated (preventable) ADE	harm-related (injuries)	prescription-level
	Prescribing errors (all types) Near misses Rule violations	process-related (error rate)	prescription-level
	Alerts	process-related (alert rate)	prescription-level
	Overrides	process-related (response rate)	alert-level
Andersson et al., 2013 [40]	**Primary Outcome: (Severe) DDI**	**process-related (error rate)**	**prescription-level**
Field et al., 2009 [41]	PIM (list of target medications)	process-related (error rate)	patient-level
	Alerts	process-related (alert rate)	patient-level
	Appropriate drug orders after alert	process-related (response rate)	alert-level
Glassman et al., 2007 [42]	**Primary Outcome: ADE** *(Includes hospitalization and death)*	**harm-related (injuries, hospitalization & mortality)**	**patient-level**
	Conflicts	process-related (error rate)	patient-level
Gurwitz et al., 2008 [43]	**Primary Outcome: ADE** *(Includes falls)*	**harm-related (injuries & injury risk)**	**patient-level**
Hou et al., 2013 [44]	Initial and final dose errors	process-related (error rate)	prescription-level
	Near miss detection rate	process-related (alert rate)	prescription-level
	Acceptance rate	process-related (response rate)	alert-level
	Near miss blocking rate	process-related (response rate)	prescription-level
Humphries et al., 2007 [45]	**Primary Outcome: DDI**	**process-related (error rate)**	**prescription-level & patient-level**
Jani et al., 2008 [46]	**Primary Outcome: Prescribing errors**	process-related (error rate)	prescription-level
	Error-free patient visits	process-related (error rate)	encounter-level
Judge et al., 2006 [47]	Alerts	process-related (alert rate)	patient-level
	Appropriate actions after alert	process-related (response rate)	alert-level
Jungo et al., 2023 [48]	Falls	harm-related (injury risk)	patient-level
	Fractures	harm-related (injuries)	patient-level
	HRQoL	harm-related (HRQoL)	patient-level
	**Primary Outcome 1: PIM (Medication appropriateness)**	**process-related (error rate)**	**patient-level**
	**Primary Outcome 2: PIP (Prescribing omissions)**	**process-related (error rate)**	**patient-level**
	Prescribing recommendations	process-related (alert rate)	patient-level
	Implementation of prescribing recommendations	process-related (response rate)	patient-level & alert-level
Kahan et al., 2017 [49]	Alerts	process-related (alert rate)	encounter-level
	System access after alert Resolved alerted interactions Unchanged alerted interactions	process-related (response rate)	alert-level
	Hospitalizations	cost-related (HCRU)	patient-level
	Medication volume	cost-related (HCRU)	patient-level
	Imaging episodes	cost-related (HCRU)	patient-level
Kaushal et al., 2011 [50]	Prescribing errors Near misses Rule violations	process-related (error rate)	prescription-level
Kaushal et al., 2010 [51]	Preventable ADE	harm-related (injuries)	prescription-level
	Prescribing errors Near misses Rule violations	process-related (error rate)	prescription-level
Mazzaglia et al., 2016 [52]	**Primary Outcome: Recommended drug use**	**process-related (error rate)**	**patient-level**
	Potential DDI	process-related (error rate)	patient-level
Overhage et al., 2016 [53]	**Primary Outcome: Preventable ADE**	**harm-related (injuries)**	**patient-level**
	ADE	harm-related (injuries)	patient-level
	Potential ADE	process-related (error rate)	patient-level
Price et al., 2017 [54]	**Primary Outcome: PIP (based on STOPP criteria)**	process-related (error rate)	prescription-level
Raebel et al., 2007a [55]	**Primary Outcome: PIM (category D or X medications)**	**process-related (error rate)**	**patient-level**
	Alerts	process-related (alert rate)	patient-level
Raebel et al., 2007b [56]	**Primary Outcome: PIM (Based on Beers and Zhan criteria)**	**process-related (error rate)**	**patient-level**
	Responses to alerts	process-related (response rate)	prescription-level
Rieckert et al., 2020 [57]	**Primary Outcome: Unplanned hospital admission or death**	**harm-related (hospitalization & mortality)**	**patient-level**
	Unplanned hospital admission	harm-related (hospitalization)	patient-level
	All-cause mortality	harm-related (mortality)	patient-level
	Falls	harm-related (injury risk)	patient-level
	Fractures ADR	harm-related (injuries)	patient-level
	HRQoL	harm-related (HRQoL)	patient-level
	CDSS recommendations	process-related (alert rate)	patient-level
Schwarz et al., 2012 [58]	**Primary Outcome: Appropriate actions after alert**	**process-related (response rate)**	**alert-level & encounter-level**
	Alerted encounters	process-related (alert rate)	encounter-level
Simon et al., 2006 [59]	**Primary Outcome: PIM (list of target medications)**	**process-related (error rate)**	**patient-level**
	Alert rate	process-related (alert rate)	physician-level
Smith et al., 2006 [60]	PIM (Dispensings of nonpreferred medication) Dispensings of preferred medication	process-related (error rate)	patient-level
Steele et al., 2005 [61]	(Potential) ADE	harm-related (injuries)	patient-level
	Medication orders not completed after alert Rule-associated laboratory test orders after alert	process-related (response rate)	prescription-level
	Triggered rules Displayed alerts	process-related (alert rate)	prescription-level
Subramanian et al., 2012 [62]	Alerts	process-related (alert rate)	patient-level
	Drug and laboratory test costs	cost-related (direct)	patient-level
Tamblyn et al., 2012 [63]	**Primary Outcome: Risk of injury**	**harm-related (injury risk)**	**patient-level**
	Response to alerts	process-related (response rate)	alert-level
	PIM (list of target medications)	process-related (error rate)	patient-level
	Alerts	process-related (alert rate)	patient-level
Tamblyn et al., 2008 [64]	**Primary Outcome: Prescribing errors**	**process-related (error rate)**	**patient-level**
	Response to alerts	process-related (response rate)	alert-level
	Alerts	process-related (alert rate)	patient-level
Tamblyn et al., 2003 [65]	**Primary Outcome 1: Prescribing errors (initiation)**	**process-related (error rate)**	**encounter-level**
	**Primary Outcome 2: Prescribing errors (discontinuation)**	**process-related (error rate)**	**encounter-level**
Vanderman et al., 2017 [66]	**Primary Outcome 1: New PIM (based on Beers Criteria)**	**process-related (error rate)**	**prescription-level**
	**Primary Outcome 2: New top 10 PIM**	**process-related (error rate)**	**prescription-level**
	**Primary Outcome 3: New flagged PIM**	**process-related (error rate)**	**prescription-level**
	**Primary Outcome 4: New (non-PIM) tracer medications**	**process-related (error rate)**	**prescription-level**
Witte et al., 2019 [67]	**Primary Outcome: PIM (based on PRISCUS)**	**process-related (error rate)**	**patient-level**
	Medication adjustment after alert	process-related (response rate)	alert-level
	Medication volume	cost-related (HCRU)	patient-level
	Drug-related costs	cost-related (direct)	patient-level & prescription-level
Zillich et al., 2008 [68]	**Primary Outcome: PIM (based on Beers Criteria)**	**process-related (error rate)**	**patient-level**
	PIM (discontinuation)	process-related (error rate)	patient-level

*ADE *Adverse drug event, *ADR *Adverse drug reaction, *AOU *Assessment of underutilization, *CDSS *Clinical decision support system, *DDI *Drug-drug interaction, *HCRU *Health care resource utilization, *HrQoL *Health-related quality of Life, *MAI *Medication appropriateness index, *PIM *Potentially inappropriate medication, *PIP *Potentially inappropriate prescribing

**Table 3:** Overview and frequency of outcome levels used by included studies per outcome category and subcategory (*n* = number of studies)

	Outcome level				
Outcome (sub)category	Patient	Prescription	Alert	Encounter	Physician
**Process-related**					
Alert rate	8	3	-	2	1
Response rate	1	3	10	1	-
Error rate	14	11	-	2	-
**Harm-related**					
Injury risk (includes falls)	4	-	-	-	-
Injuries (ADE/fractures)	6	4	-	-	-
Hospitalization	2	-	-	-	-
Mortality	2	-	-	-	-
HrQoL	2	-	-	-	-
**Cost-related**					
HCRU	2	-	-	-	-
Direct costs	2	1	-	-	-

*ADE* Adverse drug event, *HCRU* Health care resource utilization, *HrQoL* Health-related Quality of Life

**Table 4:** Empirical findings of studies (primary outcomes)

Study	Primary Outcome	Significant intervention effect	No significant intervention effect
**Studies using harm-related primary outcomes**			
Glassman et al., 2007 [42]	ADE		x
Gurwitz et al., 2008 [43]	ADE		x
Overhage et al., 2016 [53]	Preventable ADE		x
Rieckert et al., 2020 [57]	Unplanned hospital admission or death		x
Tamblyn et al., 2012 [63]	Risk of injury	x	
**Studies using process-related primary outcomes**			
Andersson et al., 2013 [40]	DDI	x	
Humphries et al., 2007 [45]	DDI	x	
Jani et al., 2008 [46]	Prescribing errors	x	
Jungo et al., 2023 [48]	PIM (Medication appropriateness) PIP (Prescribing omissions)		x (Medication appropriateness and prescribing omissions)
Mazzaglia et al., 2016 [52]	Recommended drug use	x (not for all subgroups)	
Price et al., 2017 [54]	PIP (based on STOPP criteria)		x
Raebel et al., 2007a [55]	PIM (category D or X medications)	x	
Raebel et al., 2007b [56]	PIM (Based on Beers and Zhan criteria)	x	
Schwarz et al., 2012 [58]	Appropriate actions after alert		x
Simon et al., 2006 [59]	PIM (list of target medications)		x
Tamblyn et al., 2008 [64]	Prescribing errors		x
Tamblyn et al., 2003 [65]	Prescribing errors (initiation) Prescribing errors (discontinuation)	x (initiation)	x (discontinuation)
Vanderman et al., 2017 [66]	PIM (based on Beers Criteria) Top 10 PIM Flagged PIM Tracer medications	x (Top 10 PIMs and tracer medications)	x (PIM and flagged PIM)
Witte et al., 2019 [67]	PIM (based on PRISCUS)		x
Zillich et al., 2008 [68]	PIM (based on Beers Criteria)	x	

*ADE* Adverse drug event, *DDI* Drug-drug interaction, *PIM* Potentially inappropriate medication, *PIP* Potentially inappropriate prescribing

#### Computerized physician order entry

Computerized Physician Order Entry (CPOE) is defined as any system that allows health care providers to “directly place orders for medications, tests or studies into an electronic system, which then transmits the order directly to the recipient responsible for carrying out the order (e.g. the pharmacy, laboratory, or radiology department)” [27].

#### Electronic prescribing

Electronic Prescribing (e-prescribing or eRx) can be seen as a special form of CPOE [69]. It is defined as the “computer-based electronic generation, transmission and filling of a prescription” [70].

#### Clinical decision support systems

Clinical Decision Support Systems (CDSS), often integrated with CPOE [27], supply health care providers and patients themselves with “knowledge and person-specific information, intelligently filtered or presented at appropriate times” [71]. Tools may include computerized alerts and reminders, clinical guidelines, patient data reports, or diagnostic support [71].

#### Electronic health records

According to the International Organization for Standardization, electronic health records (EHR) are classified as a repository of patient data in digital form, stored and exchanged securely, and accessible by multiple authorized users that primarily aim to support continuing, efficient and quality integrated health care. There are several different types of EHR [72].

We grouped outcome measures into the three main categories identified by Seidling and Bates [32]: process-related outcomes (e.g. medication errors), harm-related outcomes (e.g. ADE), and cost-related outcomes (e.g. costs of ADE, outcomes from health economic evaluations) (Table 2). For example, we categorized healthcare resource utilization outcomes (HCRU) as cost-related and effects on health (e.g. mortality or hospitalization) as harm-related [73]. Finally, we extracted the main empirical findings (for primary outcomes) of the included studies (Table 4). Heterogeneity in reported outcomes and study designs did not allow for a meta-analysis.

### Quality assessment

The methodological quality of the included studies was assessed using the Evidence Project risk of bias tool [74], which has also been used by a similar systematic review in this field [26]. This tool was selected because it allows assessing the risk of bias for both randomized and non-randomized studies. The items include (1) cohort, (2) control or comparison group, (3) pre-post intervention data, (4) random assignment of participants to the intervention, (5) random selection of participants for assessment, (6) follow-up rate of 80% or moe, (7) comparison groups equivalent on sociodemographics, and (8) comparison groups equivalent at baseline on outcome measures referring to study design, participant representativeness, and the equivalence of comparison groups (see Supplementary Table S1, Additional File 3). Item 7 was slightly expanded by not only considering sociodemographic but also disease-related factors as potential confounding variables. The tool explicitly allows such adaptions. For each study, items 1–3 and 5 were rated as present or absent; item 4 was rated as present, absent or not applicable (n.a.); items 6–8 were rated as present, absent, n.a. or not reported (n.r.). Two reviewers made independent judgments on each of the items (DGR, DL). Disagreements (*n* = 10) between the two reviewers were resolved by consensus after discussion.

## Results

### Study selection

**Fig. 1:**
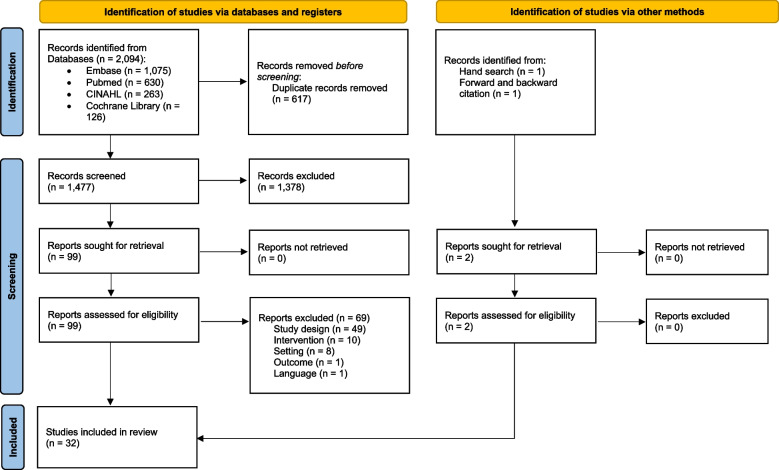
PRISMA 2020 flow diagram

The literature search identified 2,094 studies, resulting in 1,477 studies after duplicates were removed. After screening titles and abstracts, 1,378 records were excluded and 99 full-text studies were subsequently assessed for eligibility. Full-text assessment led to the exclusion of further 69 studies. Reasons for exclusion were related to a wrong study design (*n* = 49), intervention type (*n* = 10), setting (*n* = 8), outcome (*n* = 1) and language (*n* = 1). In addition to the database search, one study each was identified by forward and backward citation and by manually searching the reference lists of the included studies, respectively. Overall, we included a total of 32 studies in our review (Fig. 1).

### Study characteristics

Study characteristics and designs are presented in Table 1. The studies included 13 cluster-randomized trials (C-RCT) [41, 43, 47], 11 single-arm pre-post studies (PPS) [37, 39, 44–46, 53, 60, 61, 66–68], five non-randomized controlled trials (N-RCT) [38, 40, 49–51] and three randomized controlled trials (RCT) [42, 55, 56]. Roughly half of C-RCT studies (*n* = 6) were randomized at the physician level [48, 52, 57, 63–65], though the remainder (*n* = 7) were randomized at a higher level, either at the level of resident care units [41, 43, 47, 62] or the clinic/practice level [54, 58, 59].

The majority of studies (*n* = 24, 75%) were conducted in North America (USA/CAN) [37, 39, 41–43, 45, 47, 50, 51, 53–56, 58–66, 68] six in Europe [40, 46, 48, 52, 53, 57, 67] and two in Asia [44, 49]. Studies were predominately conducted in primary care practices/centers (PCP) [38, 40, 48, 50–54, 57, 58, 63–65, 67], in outpatient/ambulatory clinics (OC) [37, 39, 42, 44, 46, 61, 66, 68], in Health Maintenance Organizations (HMO) [45, 49, 55, 56, 59, 60] or in LTC facility settings [41, 43, 47, 62]. Sample sizes varied considerably between studies, ranging from 323 [48] to approx. 450,000 patients [60]. Study periods also varied between 4 months [55] and 57 months [45].

All but one study [48] used a CDSS in combination with other components. Most frequently, EHR [37–42, 49, 50, 53, 54, 57–68], CPOE systems [37, 39, 41, 43, 44, 47, 59–62, 66], and electronic prescribing (eRx) [37–40, 46, 50, 51, 53, 64] were used in addition to the CDSS. In addition, a subset of these or other interventional components, such as pharmacy information management systems [45, 55, 56], medication profiling software with a clinical pharmacist [42] or an educational program [59] were added. Studies also differ regarding the comparator. Most frequently, the comparator consisted of EHR [37, 39–42, 49, 54, 58–66, 68], CDSS with fewer functions [37, 39, 40, 42, 45, 50, 54, 58, 59, 63, 64, 68], CPOE systems [37, 39, 41–44, 47, 59–62, 66], paper-based prescription/information [38, 46, 51–53] or a combination of these components. Other types of software [45, 67] and eRx [50, 53, 64, 67] were also utilized for comparison.

### Methodological findings

**Fig. 2:**
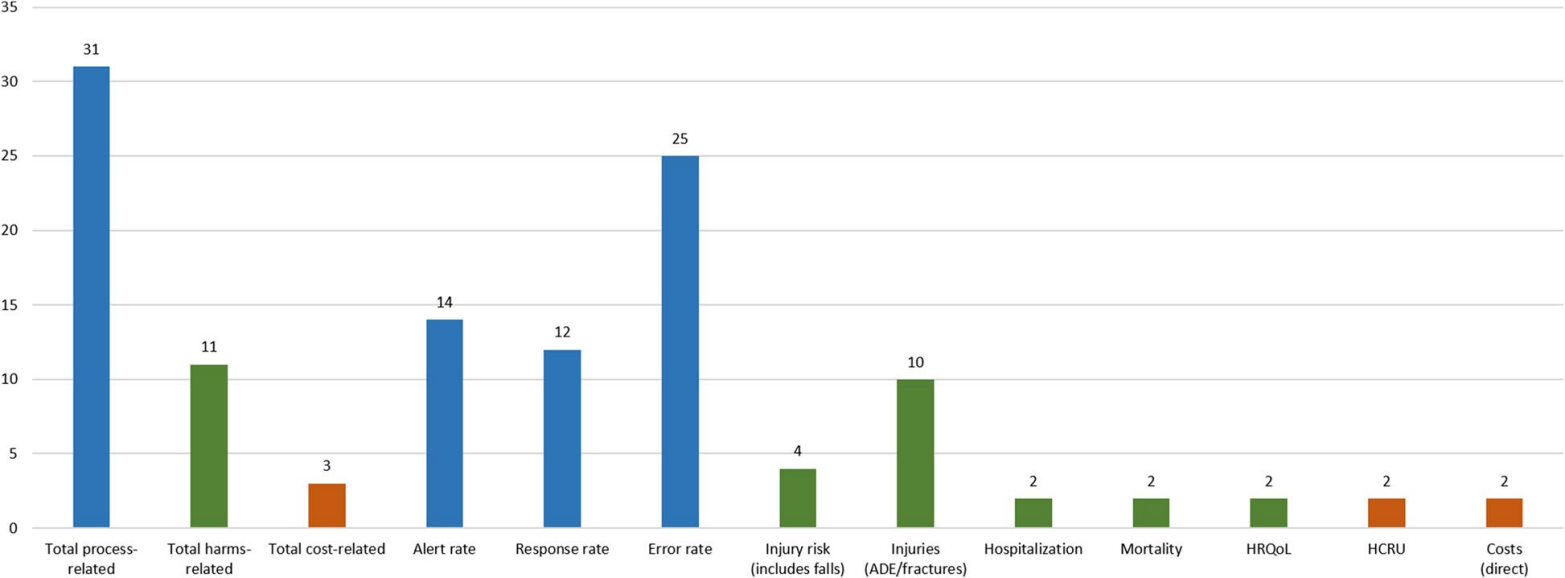
*Number of included studies using each outcome (sub-)category*

Following Seidling and Bates [32], the outcome measures used in the included studies were categorized into process-related, harm-related and cost-related outcomes. Table 2 gives an overview of the extracted outcomes for each study. Almost all studies (*n* = 31) used process-related outcomes. Of these, 18 used only process-related outcomes. Harm-related outcomes were used in 11 studies, of which one study used only harm-related outcomes. Three studies reported cost-related outcomes. Notably, no study used all three types of outcomes. In each category, we grouped the outcomes used into subcategories, shown in Fig. 2.

### Process-related outcomes

We divided the process-related outcomes used by the included studies into three subcategories, defined in more detail below. Of these subcategories, error rates were studied most frequently (*n* = 25 studies), followed by alert rates (*n* = 14) and response rates (*n* = 12). During clinical encounters involving CDSS, these three subcategories of process-related outcomes follow a temporal logic. First, alert rates measure whether CDSS alerts occurred, indicating a potential medication error in the making. Second, response rates measure whether (and/or how) prescribers react to these alerts. Finally, error rates measure the actual medication errors that reach patients.

Error rates concern the occurrence of different types of medication errors. Error rates are the most patient-relevant process-related outcomes, since medication errors may lead to ADE or other direct patient harms. As seen in Table 2, the studies used various types of errors to define error rates. These error types included potentially inappropriate medication (PIM), potentially inappropriate prescribing (PIP), drug-drug interactions (DDI), drug duplications, near misses and rule violations. A number of studies used composite outcomes combining multiple types of prescribing errors, including illegibility errors, duration errors, strength errors, directions errors, frequency errors, amount errors, dose errors, route errors, refill errors and inappropriate abbreviations. Finally, some studies did not measure the number of errors, but rather the absence of errors (such as error-free patient visits or recommended drug use); these outcomes were also categorized as error rates.

Most studies used error rates defined at the patient-level, such as the number of errors (of a given type) per patient/person/person-time, or at the prescription-level, such as the number of errors per prescription/medication/dispensing. Two studies used error rates defined at the encounter-level (the number of errors per encounter/visit).

Alert rates measure the number of alerts generated by the CDSS. Alerts do not directly impact patients and are therefore less patient-relevant than error rates, although accurate alerts that lead to appropriate responses by prescribers can prevent the occurrence of medication errors. Types of alerts included warnings (such as dose, frequency, interaction, avoid or missing information alerts) and recommendations (such as START and STOPP recommendations or dose recommendations). Most studies using alert rates defined these outcomes at the patient-level, although a smaller number of studies defined alert rates at the prescription-, encounter- or physician-level (the number of alerts per physician).

Response rates concern the ways in which prescribers respond to and interact with the CDSS and the alerts it generates. Response rates do not directly impact patients and are therefore also less patient-relevant than error rates. However, these responses do influence whether medication errors occur following alerts, thereby indirectly impacting patients. There was significant heterogeneity in the response types investigated by the included studies. These response types included implementing CDSS recommendations, resolving or overriding alerts, correcting or modifying prescriptions (including medication, dose and frequency), discontinuing mediations and other appropriate actions after alerts. Most studies using response rates defined these outcomes at the alert-level (the number of responses per alert). A smaller number of studies used response rates defined at the prescription-level or patient-level.

### Harm-related outcomes

Harm-related outcomes most frequently comprised ADE or fractures, which we grouped under injuries (*n* = 10), followed by injury risk (*n* = 4), which includes falls. Two studies each used Health-related Quality of Life (HRQoL), mortality and hospitalization (Fig. 2). Most studies used the Naranjo algorithm [75] for classifying ADE; two studies [43, 57] used other methods. Most harm-related outcomes were defined at the patient-level, although four studies defined (preventable) ADE at the prescription-level.

### Cost-related outcomes

Only three of the included studies used cost-related outcomes. Of these studies, one [49] assessed only HCRU, one [62] assessed only direct costs and one [67] assessed both HCRU and direct costs. No studies assessed indirect costs. Both studies assessing direct costs included only a small subset of these costs: Witte et al. [67] compared direct drug-related costs resulting from a difference in the observed prescription volumes between the intervention and control period, while Subramanian et al. [62] estimated the costs that would have been incurred if drug orders that triggered the alert system had actually been completed compared to the costs of the final submitted orders. One study [48] references a full health economic evaluation conducted alongside the effectiveness trial. This health economic evaluation reportedly takes into account both direct (e.g. doctor visits) and indirect (e.g. informal care) costs. However, as of our search, the corresponding paper has not yet been published and is therefore not included in this review.

Table 3 gives an overview of the outcome levels used by the included studies per outcome category and subcategory. The patient-level was the most common for all process-related outcomes except response rates, which were most commonly defined at the alert-level. Notably, response rates were also the only outcomes of any kind to be defined at the alert-level. Finally, harm- and cost-related outcomes were overwhelmingly defined at the patient-level, though some injury outcomes were also defined at the prescription-level.

### Empirical findings

Slightly more than half of the studies (*n* = 20) explicitly specified a primary outcome (Table 4), three studies specified multiple primary outcomes. Most studies (*n* = 15) used process-related primary outcomes, of which roughly half (*n* = 8) were PIM or PIP. Five studies used harm-related primary outcomes, three of which were (preventable) ADE. No study specified a cost-related primary outcome.

Half of studies with primary outcomes (*n* = 10) demonstrated a significant intervention effect for at least one primary outcome. However, only one out of five studies with harm-related primary outcomes (20%) found a significant intervention effect, compared to nine out of fifteen studies with process-related primary outcomes (60%). Of the three studies with multiple primary outcomes, two found significant intervention effects for some primary outcomes, but not for others (Table 4).

### Quality assessment

We found that at least half of the included studies demonstrated a potential risk of bias. First, half of the studies were either PPS (*n* = 11), which lack a separate control group, or N-RCT (*n* = 5), which use a non-randomized control group. In contrast, C-RCT (*n* = 13) and RCT (*n* = 3) studies, which use randomized control groups, demonstrate less risk of bias. Second, most studies that did use a (randomized or non-randomized) control group either reported problems regarding the comparability of study groups or did not address study group comparability at all. Third, most of the studies were cross-sectional (*n* = 22) instead of using a longitudinal (*n* = 10) design (see Supplementary Table S1, Additional File 3).

## Discussion

This systematic review identified and categorized outcomes used in experimental studies evaluating the effects of medication-related CDSS implemented in primary and LTC settings. We grouped outcome measures into three categories identified by Seidling and Bates [32]: harm-related, process-related and cost-related. Across the included studies, there was substantial heterogeneity with regards to study design, outcome measures and main empirical findings.

### Choosing outcome measures

Which outcomes should be used to evaluate CDSS? From a patient perspective, harm-related outcomes are most relevant. Medication-related outcomes (such as ADE) may be better suitable for evaluating the isolated health impact of CDSS than more general outcomes (such as HRQoL, hospitalization or mortality), since the latter depend on various factors besides the CDSS [76]. Nevertheless, HRQoL, hospitalization and mortality are highly patient-relevant outcomes. If possible, studies should therefore use medication-related outcomes alongside more general harm-related outcomes.

When the use of harm-related outcomes is not possible or feasible, error rates can serve as a process-related proxy for patient harm. Alert rates and response rates, however, are less suitable as proxies for patient harms. Whenever possible, studies should use harm-related primary outcomes rather than process-related proxies [76].

While process-related outcomes should not replace direct measures of patient harms, they provide important information about system activity and should therefore be included as outcomes in CDSS evaluations. For example, a high alert rate and low response rate may indicate alert fatigue, suggesting improvements aimed at usability and user experience [77]. In contrast, a low alert rate, high response rate and high error rate may indicate that while prescribers are willing to use the system, not enough alerts are generated to meaningfully improve patient outcomes. To comprehensively assess CDSS activity, studies should use error, response and alert rates.

Finally, while cost-related outcomes are not directly patient-relevant, they represent important secondary outcomes and should therefore be included in CDSS evaluations. The health economic impacts of novel interventions are increasingly important for resource allocation decisions [78]. However, the cost-related evaluation of CDSS remains a challenging task, as these complex digital health interventions usually influence the medication process in several ways [32]. Furthermore, using secondary data on direct and indirect costs for economic evaluations is not always feasible and primary cost-related data may be difficult to collect.

Besides direct intervention costs (such as those related to the implementation), studies should also include indirect intervention costs (such as time spent training with new software). However, these indirect costs are difficult to measure and are thus often not considered [79]. For example, Donovan et al. show that the implementation costs of hospital-based CDSS are rarely reported and the methods used to measure and value such costs are often not well described [80]. Thus, intervention costs, as well as costs that may have occurred in other (health care) sectors, are often not considered in economic evaluations of CDSS [81]. Since the quality of the current health economic literature on health information technology in medication management is poor [81], future studies should follow established standards of health economic evaluations [78, 82, 83]. Additionally, since the economic impacts of improved medication safety may occur on different levels, economic evaluations of CDSS should take into account not only the payers’ perspective, but also financial effects at the provider level.

To summarize: CDSS evaluations should include multiple outcomes from each of the three outcome categories [32, 76]. However, we found that none of the included studies conducted a comprehensive evaluation of all three outcome categories. Furthermore, two-thirds of studies did not consider any harm-related outcomes. Those studies that did use harm-related outcomes mostly used ADE or other injuries; very few used morbidity or hospitalization. Although process-related outcomes were by far the most used outcomes, this is mostly due to the large number of studies using error rates. In contrast, response rates and alert rates were used less commonly, making it difficult to fully investigate and interpret CDSS activity and use. Finally, only three studies used cost-related outcomes. This finding is consistent with the sparse and conflicting evidence regarding the financial impact and cost-effectiveness of CDSS [16, 81, 84]. The studies that used cost-related outcomes included only a small subset of direct costs and did not consider indirect costs.

### Defining outcome measures

We have seen that the included studies differ in the outcome categories they use. However, studies also differ in their definition and operationalization of outcomes even within categories (and subcategories).

While mortality and hospitalization are easily measured standardized outcomes, other harm-related outcomes (such as injuries) may be defined and operationalized in various ways, limiting the comparability of harm-related results between studies. Cost-related outcomes were only considered in three studies, which used significantly different (and therefore non-comparable) approaches.

Differences in outcome definition and operationalization between studies were most pronounced for process-related outcomes. First, these outcomes measured the occurrence of a number of different types of errors, responses, and alerts. For example, an error rate may refer to the number of PIM or the number of DDI. Second, these outcomes can be defined at different levels, including patient-level, encounter-level, prescription-level or alert-level. For example, an error rate may refer to the number of errors per prescription or the number of errors per patient-month. These differences in outcome definitions are in line with the literature: a review by Rinke et al. [85] also found differences in outcome definition and operationalization for evaluations of interventions to reduce paediatric medication errors.

Due to these differences in outcome definition, comparing results between studies can be difficult or even impossible [85], even if studies use the same outcome categories. Therefore, future research should work toward consensus definitions for key outcomes. This could increase the efficiency of evidence synthesis and reduce the risk of duplicated research efforts, thereby accelerating the improvement of care [86]. When agreed-upon definitions are unavailable, researchers can increase the comparability of their results by reporting multiple outcome definitions.

Importantly, this does not imply that all CDSS evaluation research should use a one-size-fits-all approach. Different healthcare systems, care settings, study populations, or CDSS types may give rise to different research questions, which will likely require the use of different outcomes and definitions. For example, an evaluation of a novel CDSS introduced in an LTC setting with a history of inappropriate medications may use a PIM/PIP-based error rate, while an evaluation of an existing primary care CDSS which has recently been upgraded to generate dosage alerts may instead measure the rate of dosage errors. However, studies with similar research questions concerning similar settings and populations should still strive to use comparable outcome definitions, when possible.

Finally, researchers should carefully consider at which level they define their outcomes. For many types of error rates, the prescription-level may be most appropriate. For example, the number of errors per prescription (or per encounter) reflects the total opportunities for errors more accurately than the number of errors per patient or per patient-month [85]. Similarly, it may be more appropriate to define response rates at the alert-level, rather than the prescription-level. As discussed above, the most appropriate outcome definition will depend on the context and specific research question.

### Reducing the risk of bias

But even if the included studies had used a wider variety of outcomes from all outcome categories, with agreed-upon definitions and standardized operationalizations for each outcome, many studies would still have exhibited a risk of bias due to their study design and other methodological problems. In particular, most studies were cross-sectional designs without a sufficient follow-up period, many studies were not randomized or not controlled and most controlled studies did not demonstrate study group comparability. Finally, many studies did not specify a primary outcome, and only 12 studies reported power calculations.

To reduce the risk of bias, future research should rely on well-designed (cluster) RCTs including a sufficient follow-up period; study group comparability should be assessed and reported. Whenever possible, studies should be longitudinal rather than cross-sectional. Finally, studies should explicitly specify a clear (preferably harm-related) primary outcome and should perform and report sample size and power calculations for this outcome.

### Empirical findings

Only 20 out of 32 included studies explicitly specified a clear primary outcome and, of these, only five studies used harm-related primary outcomes. While half of all studies with primary outcomes demonstrated a significant intervention effect, most studies finding significant effects did so for process-related primary outcomes. This result is in line with current research demonstrating significant intervention effects when using process-related outcomes [18–22]. In contrast, only one study found a significant intervention effect for a harm-related primary outcome. Overall, our results agree with prior reviews finding that the effectiveness of CDSS for medication safety in primary care [27–29] and LTC settings [29–31] remains inconsistent and future research on the harm-related effects of medication-related CDSS is needed.

To generate stronger evidence on the effectiveness of CDSS, future studies should follow the methodological recommendations outlined above. Furthermore, additional research should take place in LTC settings, as this setting was underrepresented in the included studies. Finally, insights from research using process-related outcomes to study CDSS activity should be used to improve on the design and functionality of future CDSS. While uptake levels are rarely reported in CDSS evaluations, available evidence indicates that uptake is low [87]. In addition to alert fatigue, high override rates are an increasingly important problem for CDSS interventions [88, 89]. If these overrides are inappropriate, they can lead to medication errors, patient harms and increased costs [90]. Comprehensive CDSS evaluations using a variety of outcomes and outcome categories are therefore needed to identify and remove barriers to user acceptance of CDSS.

### Limitations

Compared to a recent review [26], we expanded our scope by including the LTC setting and focusing primarily on methodological aspects and outcomes used in CDSS evaluations. However, our systematic review still has several limitations. First, relevant studies that have not been indexed in the searched databases might be missing from this review, although we followed an extensive search strategy, including hand search and automated citation tools alongside the search of multiple databases. Second, due to the methodological heterogeneity of the included studies, we only compared whether or not studies found a significant effect for their primary outcome and did not compare levels of significance or effect sizes. We also did not consider outcomes related to user acceptance of CDSS. Finally, a scoping review may also have been an appropriate method for addressing our primary (methodological) aim, although the lines between these types of reviews are often blurred [91]. However, due to our secondary (empirical) aim and our performance of a risk of bias assessment, we decided to conduct a full systematic review according to the PRISMA, rather than PRIMSA Extension for Scoping Reviews [92], guidelines.

The included studies vary in terms of applied interventions and comparisons. Some studies compared the CDSS intervention to non-automated IT systems, while other studies used handwritten or paper-based prescription forms as a comparison. Consequently, the applied interventions and comparisons are not comparable, which could also have an influence on the differences in outcome measures and operationalizations. For example, comparing CDSS to other IT systems rather than handwritten prescriptions may allow alert rates or response rates to be calculated for both the intervention and control groups.

Furthermore, since 75% of the studies were from North America, the generalizability of the studies to other regions may be limited. Finally, the included studies’ high risk of bias (particularly for PPS and N-RCT studies), their lack of clearly specified primary outcomes and their weak reporting of sample sizes need to be considered when drawing conclusions from study results. Despite these limitations, our results give rise to a number of key recommendations for future studies researching the effect of CDSS on medication safety, summarized in Table 5.

**Table 5:** Recommendations for research on medication safety-related CDSS effectiveness

**Choosing Outcome Measures**
▪ Studies should use a range of harm-related, process-related, and cost-related outcomes
▪ Studies should use harms-related primary outcomes
▪ Harm-related outcomes should include medication-related and general outcomes
▪ Studies should avoid using process-related outcomes as proxies for patient harms
▪ Process-related outcomes should include error rates, response rates and alert rates
▪ Cost-related outcomes should include direct and indirect costs
▪ Studies using cost-related outcomes should consider the payer and provider perspectives
**Defining Outcome Measures**
▪ Studies should use agreed-upon outcome definitions
▪ Outcomes should be defined at the appropriate level (e.g. patient-level vs. prescription-level)
**Reducing the Risk of Bias**
▪ Studies should use (cluster) RCT designs rather than PPS or N-RCT designs
▪ Studies should assess and report study group comparability
▪ Studies should use longitudinal, rather than cross-sectional designs
▪ Studies should explicitly specify their primary outcomes and calculate sample size/power

## Conclusions

Our primary aim in this review was to summarize and categorize the outcome measures used in CDSS evaluation studies. Furthermore, we assessed the methodological quality of these studies and compared their key findings.

Although a variety of studies have evaluated the effectiveness of CDSS, we found that these studies face a number of (methodological) problems that limit the generalizability of their results. In particular, no studies used a comprehensive set of harm-related, process-related and cost-related outcomes. Definitions and operationalizations of outcomes varied widely between studies, complicating comparisons and limiting the possibility of evidence synthesis. Furthermore, a number of studies were not controlled, lacked randomization or did not demonstrate the comparability of study groups. Only 63% of studies explicitly specified a primary outcome. Of these, half found a significant intervention effect.

Overall, evidence on CDSS effectiveness is mixed and evidence synthesis remains difficult due to methodological concerns and inconsistent outcome definitions. Additional high-quality studies using a wider array of harm-, process- and cost-related outcomes are needed to close this evidence gap and increase the availability of effective CDSS in primary care and LTC.

### Supplementary Information

Supplementary Material 1.

Supplementary Material 2.

Supplementary Material 3.

## Data Availability

No datasets were generated or analysed during the current study.

## Abbreviations

ADE: Adverse drug event
ADR: Adverse drug reaction
CDSS: Computerized decision support system
CPOE: Computerized provider order entry
C-RCT: Cluster-randomized controlled trial
DDI: Drug-drug interactions
EHR: Electronic health record
eRx: Electronic prescribing
HCRU: Healthcare resource utilization
HMO: Health Maintenance Organization
HRQoL: Health-related Quality of Life
LTC: Long-term care
N-RCT: Non-randomized controlled trial
n.a: Not applicable
OC: Outpatient/ambulatory clinic
PCP: Primary care practice/centers
PIM: Potentially inappropriate medication
PIP: Potentially inappropriate prescribing
PPS: Pre-post study
RCT: Randomized controlled trial
