# Supplementary material for: How intervention studies measure the effectiveness of medication safety-related clinical decision support systems in primary and long-term care: a systematic review
Source: BMC Med Inform Decis Mak. 2024 Jul 4;24:188. doi: 10.1186/s12911-024-02596-y (PMC11225126; doi:10.1186/s12911-024-02596-y)
Supplement: Supplementary file 2 — Supplementary Material 2. [file 12911_2024_2596_MOESM2_ESM.docx]

**Table S1.** Search strategy

| **PubMed via Medline** | |
| --- | --- |
| Digital character of Intervention | |
| #1 | (((((("computerized") OR "computerised") OR "computer based") OR "digital") OR "digitalized") OR "digitalised") OR "electronic" |
| Type of intervention | |
| #2 | (((((((((((((((("decision support") OR "decision making support") OR "cds") OR "dss") OR "cdss") OR "ccds") OR "medication alert") OR "medication alerts") OR "drug alert") OR "drug alerts") OR "safety alert") OR "safety alerts") OR "interaction alert") OR "interaction alerts") OR "alert system") OR "alert systems") OR "decision support systems, clinical"[MeSH Terms] |
| Purpose of intervention | |
| #3 | (((((((((((((((((((((((((((((((((((((("medication safety") OR "prescribing safety") OR "prescription safety") OR "quality of prescribing") OR "prescribing quality") OR "prescription quality") OR "safe prescribing") OR "safe prescription") OR "safe prescriptions") OR "unsafe prescribing") OR "appropriate prescribing") OR "appropriate prescription") OR "appropriate prescriptions") OR "inappropriate prescribing") OR "inappropriate prescription") OR "inappropriate prescriptions") OR "appropriate medication") OR "appropriate medications") OR "inappropriate medication") OR "inappropriate medications") OR "patient safety") OR "adverse outcome") OR "adverse outcomes") OR "adverse effect") OR "adverse effects") OR "adverse event") OR "adverse events") OR "adverse drug event") OR "adverse drug events") OR "ade") OR "interacting drugs") OR "drug interaction") OR "drug interactions") OR "medication error") OR "medication errors") OR "prescribing error") OR "prescribing errors") OR "prescription error") OR "prescription errors" |
| Setting | |
| #4 | (((((((((((((((((((((((((("primary health care") OR "primary healthcare") OR "primary care") OR "ambulatory") OR "ambulant") OR "outpatient") OR "general practice") OR "general practices") OR "general practitioner") OR "general practitioners") OR "family practice") OR "family practices") OR "family practitioner") OR "family practitioners") OR "family physician") OR "family physicians") OR "long-term care") OR "care home") OR "care homes") OR "care facility") OR "care facilities") OR "residential home") OR "residential homes") OR "residential care") OR "resident care") OR "primary health care"[MeSH Terms]) OR "ambulatory care"[MeSH Terms] |
| #5 | #1 AND #2 AND #3 AND #4 |
| **EMBASE via Elsevier** | |
| (computerized OR computerised OR 'computer based' OR digital OR digitalized OR digitalised OR electronic) AND ('decision support' OR 'decision making support' OR cds OR dss OR cdss OR ccds OR 'medication alert' OR 'medication alerts' OR 'drug alert' OR 'drug alerts' OR 'safety alert' OR 'safety alerts' OR 'interaction alert' OR 'interaction alerts' OR 'alert system' OR 'alert systems' OR 'decision support systems, clinical'/exp) AND ('medication safety' OR 'prescribing safety' OR 'prescription safety' OR 'quality of prescribing' OR 'prescribing quality' OR 'prescription quality' OR 'safe prescribing' OR 'safe prescription' OR 'safe prescriptions' OR 'unsafe prescribing' OR 'appropriate prescribing' OR 'appropriate prescription' OR 'appropriate prescriptions' OR 'inappropriate prescribing' OR 'inappropriate prescription' OR 'inappropriate prescriptions' OR 'appropriate medication' OR 'appropriate medications' OR 'inappropriate medication' OR 'inappropriate medications' OR 'patient safety' OR 'adverse outcome' OR 'adverse outcomes' OR 'adverse effect' OR 'adverse effects' OR 'adverse event' OR 'adverse events' OR 'adverse drug event' OR 'adverse drug events' OR ade OR 'interacting drugs' OR 'drug interaction' OR 'drug interactions' OR 'medication error' OR 'medication errors' OR 'prescribing error' OR 'prescribing errors' OR 'prescription error' OR 'prescription errors') AND ('primary health care' OR 'primary healthcare' OR 'primary care' OR ambulatory OR ambulant OR outpatient OR 'general practice' OR 'general practices' OR 'general practitioner' OR 'general practitioners' OR 'family practice' OR 'family practices' OR 'family practitioner' OR 'family practitioners' OR 'family physician' OR 'family physicians' OR 'long-term care' OR 'care home' OR 'care homes' OR 'care facility' OR 'care facilities' OR 'residential home' OR 'residential homes' OR 'residential care' OR 'resident care' OR 'primary health care'/exp OR 'ambulatory care'/exp) | |
| **CINAHL via EBSCOhost** | |
| (((((((((computerized ) OR computerised ) OR "computer based" ) OR digital ) OR digitalized ) OR digitalised ) OR electronic ) AND ((((((((((((((((("decision support" ) OR "decision making support" ) OR cds ) OR dss ) OR cdss ) OR ccds ) OR "medication alert" ) OR "medication alerts" ) OR "drug alert" ) OR "drug alerts" ) OR "safety alert" ) OR "safety alerts" ) OR "interaction alert" ) OR "interaction alerts" ) OR "alert system" ) OR "alert systems" ) OR (MH "decision support systems, clinical+"))) AND ((((((((((((((((((((((((((((((((((((((("medication safety" ) OR "prescribing safety" ) OR "prescription safety" ) OR "quality of prescribing" ) OR "prescribing quality" ) OR "prescription quality" ) OR "safe prescribing" ) OR "safe prescription" ) OR "safe prescriptions" ) OR "unsafe prescribing" ) OR "appropriate prescribing" ) OR "appropriate prescription" ) OR "appropriate prescriptions" ) OR "inappropriate prescribing" ) OR "inappropriate prescription" ) OR "inappropriate prescriptions" ) OR "appropriate medication" ) OR "appropriate medications" ) OR "inappropriate medication" ) OR "inappropriate medications" ) OR "patient safety" ) OR "adverse outcome" ) OR "adverse outcomes" ) OR "adverse effect" ) OR "adverse effects" ) OR "adverse event" ) OR "adverse events" ) OR "adverse drug event" ) OR "adverse drug events" ) OR ade ) OR "interacting drugs" ) OR "drug interaction" ) OR "drug interactions" ) OR "medication error" ) OR "medication errors" ) OR "prescribing error" ) OR "prescribing errors" ) OR "prescription error" ) OR "prescription errors" )) AND ((((((((((((((((((((((((((("primary health care" ) OR "primary healthcare" ) OR "primary care" ) OR ambulatory ) OR ambulant ) OR outpatient ) OR "general practice" ) OR "general practices" ) OR "general practitioner" ) OR "general practitioners" ) OR "family practice" ) OR "family practices" ) OR "family practitioner" ) OR "family practitioners" ) OR "family physician" ) OR "family physicians" ) OR "long-term care" ) OR "care home" ) OR "care homes" ) OR "care facility" ) OR "care facilities" ) OR "residential home" ) OR "residential homes" ) OR "residential care" ) OR "resident care" ) OR (MH "primary health care+")) OR (MH "ambulatory care+")) | |
| **Cochrane Library** | |
| (((((((((computerized ) OR computerised ) OR "computer based" ) OR digital ) OR digitalized ) OR digitalised ) OR electronic ) AND ((((((((((((((((("decision support" ) OR "decision making support" ) OR cds ) OR dss ) OR cdss ) OR ccds ) OR "medication alert" ) OR "medication alerts" ) OR "drug alert" ) OR "drug alerts" ) OR "safety alert" ) OR "safety alerts" ) OR "interaction alert" ) OR "interaction alerts" ) OR "alert system" ) OR "alert systems" ) OR [mh "decision support systems, clinical"])) AND ((((((((((((((((((((((((((((((((((((((("medication safety" ) OR "prescribing safety" ) OR "prescription safety" ) OR "quality of prescribing" ) OR "prescribing quality" ) OR "prescription quality" ) OR "safe prescribing" ) OR "safe prescription" ) OR "safe prescriptions" ) OR "unsafe prescribing" ) OR "appropriate prescribing" ) OR "appropriate prescription" ) OR "appropriate prescriptions" ) OR "inappropriate prescribing" ) OR "inappropriate prescription" ) OR "inappropriate prescriptions" ) OR "appropriate medication" ) OR "appropriate medications" ) OR "inappropriate medication" ) OR "inappropriate medications" ) OR "patient safety" ) OR "adverse outcome" ) OR "adverse outcomes" ) OR "adverse effect" ) OR "adverse effects" ) OR "adverse event" ) OR "adverse events" ) OR "adverse drug event" ) OR "adverse drug events" ) OR ade ) OR "interacting drugs" ) OR "drug interaction" ) OR "drug interactions" ) OR "medication error" ) OR "medication errors" ) OR "prescribing error" ) OR "prescribing errors" ) OR "prescription error" ) OR "prescription errors" )) AND ((((((((((((((((((((((((((("primary health care" ) OR "primary healthcare" ) OR "primary care" ) OR ambulatory ) OR ambulant ) OR outpatient ) OR "general practice" ) OR "general practices" ) OR "general practitioner" ) OR "general practitioners" ) OR "family practice" ) OR "family practices" ) OR "family practitioner" ) OR "family practitioners" ) OR "family physician" ) OR "family physicians" ) OR "long-term care" ) OR "care home" ) OR "care homes" ) OR "care facility" ) OR "care facilities" ) OR "residential home" ) OR "residential homes" ) OR "residential care" ) OR "resident care" ) OR [mh "primary health care"]) OR [mh "ambulatory care"]) | |

**Table S2.** Eligibility criteria

|  | **Inclusion** | **Exclusion** |
| --- | --- | --- |
| **Patients** | - Outpatient/primary care - long-term care | - Inpatient care, acute care, emergency care |
| **Intervention** | - Computerized CDSS automatically intervening in the medication process to improve medication safety | - Non-automated intervention - Intervention with a simple reminder function - Intervention including only electronic prescribing (eRx) - Intervention limited to a single field of drug (e.g. antimicrobial stewardship, specific psychotics) or indication - Intervention targeting solely promotion of adherence |
| **Comparison** | - Any other mode of care |  |
| **Outcome** | - Outcomes related to medication safety - Any patient-related and cost-related outcomes | - Outcomes related to healthcare providers attitude or acceptance regarding CDSS - Performance/quality indicators of CDSS (e.g. sensitivity, specifity) |
| **Study type** | - Primary interventional study (experimental) | - Observational study - Study protocols - Editorials/commentaries - Reviews |
| **Language** | - English and German | - Any other language |
