# Supplementary material for: How intervention studies measure the effectiveness of medication safety-related clinical decision support systems in primary and long-term care: a systematic review
Source: BMC Med Inform Decis Mak. 2024 Jul 4;24:188. doi: 10.1186/s12911-024-02596-y (PMC11225126; doi:10.1186/s12911-024-02596-y)
Supplement: Supplementary file 3 — Supplementary Material 3. [file 12911_2024_2596_MOESM3_ESM.docx]

| **Table S1. Quality Assessment using the Evidence Projekt risk of bias tool** | | | | | | | | |
| --- | --- | --- | --- | --- | --- | --- | --- | --- |
| **Study** | **Cohort (1)** | **Control or comparison group (2)** | **Pre/post intervention data (3)** | **Random assignment of participants to the intervention (4)** | **Random selection of participants for assessment (5)** | **Follow-up rate of 80% or more (6)** | **Comparison groups equivalent on sociodemographics (7)** | **Comparison groups equivalent at baseline on disclosure (8)** |
| Abramson et al., 2011a [66] | No | Yes | Yes | No | No | NA | Yes | Yes |
| Abramson et al., 2011b [55] | No | No | Yes | NA | No | NA | NA | NA |
| Abramson et al., 2013 [56] | No | No | Yes | NA | No | NA | NA | NA |
| Andersson et al., 2013 [67] | No | Yes | Yes | No | No | NA | NR | No |
| Field et al., 2009 [42] | No | Yes | No | Yes | No | NA | No | NR |
| Glassman et al., 2007 [71] | Yes | Yes | Yes | Yes | No | NR | No | NR |
| Gurwitz et al., 2008 [43] | Yes | Yes | No | Yes | No | NR | NR | NR |
| Hou et al., 2013 [57] | No | No | Yes | NA | No | NA | NA | NA |
| Humphries et al., 2007 [58] | No | No | Yes | NA | No | NA | NA | NA |
| Jani et al., 2008, UK [59] | No | No | Yes | NA | No | NA | NA | NA |
| Judge et al., 2006 [44] | Yes | Yes | No | Yes | No | NR | NR | NR |
| Jungo et al., 2023 [45] | Yes | Yes | Yes | Yes | Yes | Yes | NR | No |
| Kahan et al., 2017 [68] | No | Yes | Yes | No | No | NA | No | NR |
| Kaushal et al., 2011 [69] | No | Yes | Yes | No | No | NA | No | No |
| Kaushal et al., 2010 [70] | No | Yes | Yes | No | No | NA | No | Yes |
| Mazzaglia et al., 2016 [46] | Yes | Yes | Yes | Yes | No | NR | No | NR |
| Overhage et al., 2016 [60] | No | No | Yes | NA | No | NA | NA | NA |
| Price et al., 2017 [47] | No | Yes | Yes | Yes | No | NA | No | No |
| Raebel et al., 2007a [72] | Yes | Yes | No | Yes | No | NR | No | NR |
| Raebel et al., 2007b [73] | Yes | Yes | No | Yes | No | NR | Yes | NR |
| Rieckert et al., 2020 [48] | Yes | Yes | Yes | Yes | No | No | NR | NR |
| Schwarz et al., 2012 [49] | No | Yes | Yes | Yes | No | NA | Yes | NR |
| Simon et al., 2006 [50] | No | Yes | Yes | Yes | No | NA | No | NR |
| Smith et al., 2006 [61] | No | No | Yes | NA | No | NA | NA | NA |
| Steele et al., 2005 [62] | No | No | Yes | NA | No | NA | NA | NA |
| Subramanian et al., 2012 [51] | No | Yes | No | Yes | No | NA | NR | NR |
| Tamblyn et al., 2012 [52] | Yes | Yes | Yes | Yes | No | Yes | NR | NR |
| Tamblyn et al., 2008 [54] | No | Yes | Yes | Yes | No | NA | NR | NR |
| Tamblyn et al., 2003 [53] | Yes | Yes | Yes | Yes | No | No | NR | NR |
| Vanderman et al., 2017 [63] | No | No | Yes | NA | No | NA | NA | NA |
| Witte et al., 2019 [64] | No | No | Yes | NA | No | NA | NA | NA |
| Zillich et al., 2008 [65] | No | No | Yes | NA | No | NA | NA | NA |
| NR = not reported, NA = not applicable  Item assessment: Items 1-3,5 = Yes, No; Item 4: Yes, No, NA; Items 6-8: Yes, No, NA, NR | | | | | | | | |
